# Non-Hodgkin’s Lymphoma of the synovium discovered in total knee arthroplasty: a case report

**DOI:** 10.1186/1756-0500-5-449

**Published:** 2012-08-20

**Authors:** Jetze Visser, Vincent JJF Busch, Ineke M de Kievit-van der Heijden, Arno M ten Ham

**Affiliations:** 1 Sint Maartenskliniek, Department of Orthopaedics, Hengstdal 3, PO Box 9011, 6500GM, Nijmegen, The Netherlands; 2 Canisius Wilhelmina Hospital, Department of Pathology, Nijmegen, The Netherlands

**Keywords:** Non-Hodgkin's lymphoma, Total knee arthroplasty, Rheumatoid arthritis

## Abstract

**Background:**

Musculoskeletal involvement occurs in 25% of patients with non-Hodgkin’s lymphoma (NHL). Primary lymphoma in the joint is rare. It can present as a bone lesion or as atypical soft tissue proliferation. NHL has an increased incidence in patients with autoimmune rheumatic diseases.

**Case presentation:**

We present a case in which non-Hodgkin’s lymphoma was found coincidentally in the synovium during knee joint replacement surgery in a 69-year old woman with rheumatoid arthritis. Pigmented, vitreous tissue was resected, which turned out to be a diffuse large B-cell lymphoma after histological examination. The coincidental intraoperative finding of intra-articular non-Hodgkin’s lymphoma was reported twice before, presenting as synovial proliferation in elbow and shoulder surgery. In a few other cases non-Hodgkin’s lymphoma presented most often in the knee, as a bone lesion or, when soft tissue was involved, as arthritis.

**Conclusion:**

Non-Hodgkin’s lymphoma should be considered in patients with autoimmune rheumatic diseases. In case of persistent arthritis, non-respondent to anti-inflammatory drugs, a biopsy might be warranted. Moreover, when arthroscopy or arthrotomy is planned, any atypical tissue should be sent for histological analysis. Early diagnosis of NHL can contribute to improved outcome of its rapidly developing treatment options.

## Background

Non-Hodgkin’s Lymphoma (NHL) is a malignancy of the lymphatic system of uncontrolled proliferation of B- or T-lymphocytes. The musculoskeletal system is affected in 5-25% of the patients [1-4]. Musculoskeletal involvement of NHL has been reported before as a primary bone lesion or as intra-articular soft tissue proliferation with arthritis as presenting symptom. We present a case in which atypical soft tissue found during a routine knee arthroplasty led to the diagnosis systemic NHL.

## Case presentation

A 69-year old woman was referred to our clinic with chronic left knee pain. Her walking distance was limited and she complained of joint stiffness.

### Medical History

After a sports trauma 40 years ago, the patient underwent a lateral meniscectomy of the left knee. Two loose tissue parts were removed; histological analysis showed synovial tissue with chronic inflammation possibly indicating rheumatoid arthritis (RA). The following decades she had internal and rheumatological examinations for multiple joint pain and general body weakness. Multiple joint osteoarthritis and rheumatoid factor (RF)-negative RA were diagnosed. In 1997 a total knee arthroplasty was performed on the right side. In 2006 the patient consulted a rheumatologist for chronic fatigue, pain in the left knee, elbow and both wrists and feet. Further laboratory and radiographic investigation yielded no other diagnosis than RF-negative RA.

### Examination

**Figure 1:**
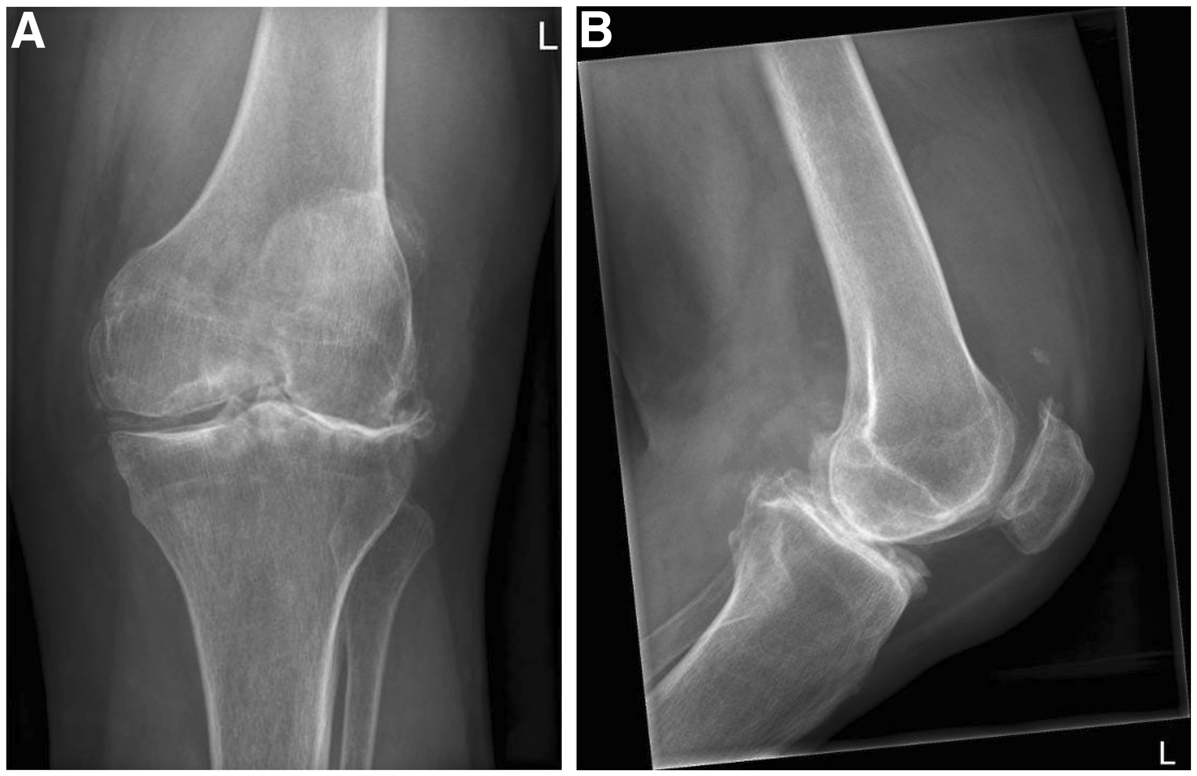
**An X-ray of the left knee showed severe osteoarthritis with evident lateral joint space narrowing, bone sclerosis, osteophytes and a calcified medial meniscus. A**. anteroposterior axis. **B**. lateral axis.

A vital woman was seen with a normal hip function. There was a correctable valgus deformity of the left leg with slight effusion of the knee and tenderness on palpation of the lateral joint space. No inflammation was seen and range of motion was normal. A conventional X-ray showed severe lateral osteoarthritis of the knee with loss of height of the lateral tibial plateau (Figure 1A,B).

### Surgery

With informed consent of the patient we decided to proceed to a total knee replacement. Intraoperatively, pigmented vitreous synovial tissue was seen in the subcutaneous tissue, which was resected and sent to the pathology department for further analysis. A total knee replacement could be performed without any complications. The patient recovered well and was discharged five days postoperatively.

### Histology

**Figure 2:**
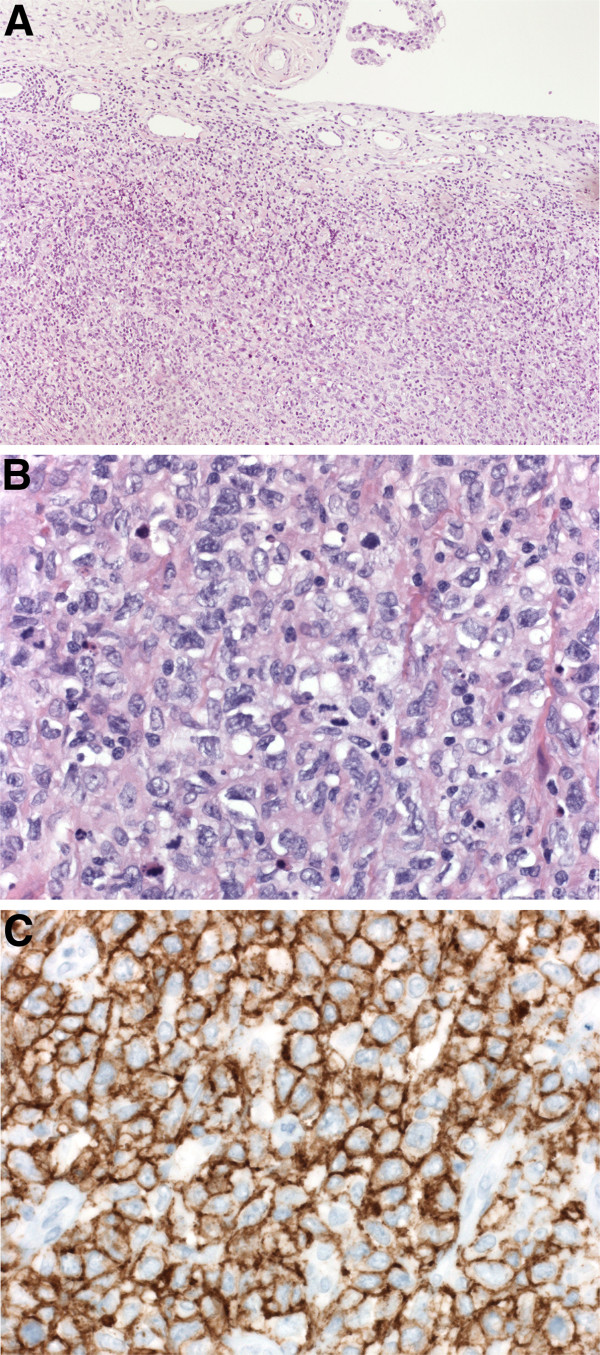
**In the synovial biopsy *(A) *a atypical lymphoid cell proliferation is present under the synovial surface (hematoxylin-eosin (HE), 2.5x) that *(B) *consists of a diffuse proliferation of large lymphoid cells (HE, 40x), showing *(C) *membranous expression of B-cell antigen CD20, which can be seen as brown deposit (40x).** This formed the histological diagnosis Non-Hodgkin Lymphoma.

Immunohistological analysis of the resected soft tissue showed a large cell lymphoid proliferation under the synovial tissue surface with expression of B-cell antigen CD-20 (Figure 2A-C). This finding matches the localization of a B-cell NHL, WHO 2008 classified as diffuse large B-cell lymphoma– not otherwise specified (DLBCL-NOS). WHO guidelines suggest an Epstein Bar Virus-negative B-cell lymphoma that develops around chronically inflamed joints in a patient with RA, as in the present case, to be classified in this category*.*

### Follow-up

Anamnesis and physical examination by an oncologist did not reveal any clues for malignancy. But a PET-CT scan showed pathologic enlarged lymph nodes along the aorta and the left iliac and inguinal vessels. Lungs and liver were clean in the scan and a bone marrow biopsy did not show signs of tumor activity. The B-cell lymphoma was staged level 2-E, therapy was started with rituximab, cyclofosfamide, vincristine, doxorubicin and prednisone (R-CHOP) combination chemotherapy in six doses.

### Bone manifestation of NHL

In 5-25% of the patients with NHL, the bone is involved, sometimes resulting in joint pain [1-4]. When this is the primary symptom, imaging techniques can raise suspicion of a malignancy. Also, bone surfaces during surgery can be suspect. A NHL was once reported in the cutting surface of the femur in a total knee arthroplasty [5]. A routine analysis of 852 retrieved femoral heads in hip arthroplasty confirms the possibility of bone involvement [6]. In 14 femoral heads a low-grade B-cell lymphoma was detected. Systemic disease was found in only two of these patients.

### Synovial manifestation of NHL

The diagnosis in this case report was based on synovial tissue analysis. This coincidental finding was reported twice before, though this was in elbow and shoulder surgery [2,7]. In a literature overview till 2006, 13 cases of intra-articular synovial manifestation of NHL were presented, of which 11 cases concerned the knee [2]. All patients presented with inflammation of the knee joint, sometimes clinically simulating RA [1,2]. In later literature two more patients were strikingly described discovering NHL with arthroscopy [3,8]. Both patients were planned for a partial meniscectomy. One patient (51 years old) had a history of gonarthritis deformans, without signs of inflammation of the knee. [8] The other patient (31 years old) had a constant knee pain and swelling [3]. Arthroscopically obtained atypical synovial tissue appeared to be a B-cell lymphoma in both cases.

Important in this case report is that patients with active RA have an increased risk of developing lymphoma [7,9-11]. A recent review showed that aggressive B-cell lymphomas, particularly the diffuse large B-cell lymphoma in the present case, are stronger associated with autoimmune rheumatic diseases than more indolent lymphomas. Although the presence of NHL was less associated with RA than with Sjögren’s syndrome and systemic lupus erythematosus [10], a 28-fold increased risk of NHL in patients with RA was found when severe damage in the knee existed in the year prior to lymphoma diagnosis [11]. Most lymphomas in this population are diffuse large B-cell lymphomas, which form an aggressive subtype of NHLs. Though with new treatment methods survival has significantly increased over the last decade [12].

The main pathophysiological mechanisms for NHL are B-cell hyperactivity and chronic inflammation [10]. Nevertheless, anti-inflammatory drugs are no treatment option for NHL [12]. Probably through its malignant character, NHL will be persistent to anti-inflammatory drugs, also in case of musculoskeletal involvement. In relation with RA, the rheumatic disease itself appears to have a larger effect on the development of lymphoma than its therapy [10]. As the presented patient had used immunosuppressive drugs (salazopyrine and leflunomide) for only two years, the contribution of drugs can be expected to be minimal. Part of the patients with a lymphoma is infected with the Epstein-Barr Virus (EBV) [9]. The presence of EBV in the lymphoma can have therapeutic consequences. Though, in the presented case no EBV-encoded RNA was found with in-situ hybridisation.

## Conclusions

To conclude we can say NHL seldom has its primary presentation in a joint. If it occurs, it presents most often in the knee as arthritis. To our knowledge we are the first to present a coincidental finding of soft tissue NHL in knee arthroplasty, without malignant bone pathology or signs of arthritis. However it is possible that the lymphoma was partly responsible for the diffuse joint pain and general body weakness this patient had for several years.

In patients with a rheumatic disease, we advice to consider NHL in the diagnostic work-up if arthritis does not respond to anti-inflammatory drugs. In these cases a biopsy may be warranted, since the risk of developing a lymphoma is significantly increased in this population. Moreover, when arthroscopy or arthrotomy is planned, any atypical tissue should be sent for histological analysis. Early diagnosis of NHL can contribute to improved outcome of its rapidly developing treatment options.

## Consent

Written informed consent was obtained from the patient for publication of this Case report and any accompanying images. A copy of the written consent is available for review by the Series Editor of this journal.

## Abbreviations

NHL: Non-Hodgkin’s Lymphoma; RF: Rheumatoid Factor; RA: Rheumatoid Arthritis; DLBCL-NOS: Diffuse large B-cell lymphoma– not otherwise specified.

## Competing interests

All four authors declare they have no competing interests. All questions underneath are answered NO by all authors.

### Financial competing interests

· In the past five years have you received reimbursements, fees, funding, or salary from an organization that may in any way gain or lose financially from the publication of this manuscript, either now or in the future? Is such an organization financing this manuscript (including the article-processing charge)? **NO**

· Do you hold any stocks or shares in an organization that may in any way gain or lose financially from the publication of this manuscript, either now or in the future? **NO**

· Do you hold or are you currently applying for any patents relating to the content of the manuscript? Have you received reimbursements, fees, funding, or salary from an organization that holds or has applied for patents relating to the content of the manuscript? **NO**

· Do you have any other financial competing interests? **NO**

### Non-financial competing interests

· Are there any non-financial competing interests (political, personal, religious, ideological, academic, intellectual, commercial or any other) to declare in relation to this manuscript? **NO**

Nijmegen, 24 April 2012

Jetze Visser, Vincent Busch, Ineke de Kievit-van der Heijden, Arno ten Ham.

## Authors’ contributions

JV and VB designed and drafted the manuscript. VB and AH did the surgical procedure. IK did the histological analysis and contributed to the manuscript regarding the pathological sections. AH initiated this case report and helped to draft the manuscript. All authors read and approved the final manuscript.

## Author details

^1^Sint Maartenskliniek, Department of Orthopaedics, Hengstdal 3, PO Box 9011, 6500GM Nijmegen, The Netherlands. ^2^Canisius Wilhelmina Hospital, Department of Pathology, Nijmegen, The Netherlands.
